# Rural–urban difference in blood pressure measurement frequency among elderly with hypertension: a cross-sectional study in Shandong, China

**DOI:** 10.1186/s41043-018-0155-z

**Published:** 2018-11-22

**Authors:** Qian Wang, Lingzhong Xu, Long Sun, Jiajia Li, Wenzhe Qin, Gan Ding, Jiao Zhang, Jing Zhu, Su Xie, Zihang Yu, Chengchao Zhou

**Affiliations:** 1 School of Public Health, Shandong University, Jinan, 250012 China; 2 Collaborative Innovation Center of Social Risks Governance in Health, School of Public health, Fudan University, Shanghai, 200032 China

**Keywords:** Blood pressure measurement, Elderly, Rural–urban difference, China

## Abstract

**Background:**

Blood pressure measurement is the first step in preventing and controlling hypertension. The objective of this study is to examine the rural–urban difference towards blood pressure measurement among elderly with hypertension.

**Methods:**

A total of 2007 elderly (65+) were selected from the fifth Health Service Survey of Shandong Province in 2013. A standardized questionnaire was used to investigate the demographic characters, socioeconomic status, self-rated health, and blood pressure related index. Three logistic regression models were used to examine the difference in blood pressure measurement between rural and urban elderly. Unadjusted and adjusted logistic regression models were used to explore the associated factors of blood pressure measurement in both rural areas and urban areas.

**Results:**

The prevalence of weekly blood pressure measurement in urban elderly was higher than that in rural elderly (63.9% vs 34.3%). The rural elderly had an odds ratio (OR) for weekly blood pressure measurement of 0.467 (95%CI = 0.380–0.575) compared with urban elderly. Binary logistic regression analysis showed that medication frequency and accepting health care professionals’ guidance were common associated factors of blood pressure measurement among both rural and urban elderly; personal income was unique associated factor of blood pressure measurement among rural elderly; marital status, education level, self-rated health, and blood pressure level currently were unique associated factors of blood pressure measurement among urban elderly.

**Conclusions:**

There is a big difference in blood pressure measurement between rural and urban elderly. Interventions targeting identified at-risk subgroups, especially for those rural elderly, should be made to reduce such a gap.

## Background

Hypertension is one of the most common risk factors for cardiovascular disease [1]. It already affects more than one billion people worldwide, leading to heart attacks and strokes [2]. Complications from hypertension account for 9.4 million deaths worldwide every year [3]. In China, about 2.1 million total cardiovascular deaths and 1.2 million premature cardiovascular deaths were attributable to hypertension every year [4].

A population-based screening project in 2017 that enrolled around 1.7 million adults from all 31 provinces in mainland China showed that 44.7% of the sample had hypertension [5].

Fortunately, hypertension can be prevented. Detecting high blood pressure is the first step in preventing and controlling it. Acting Director of the WHO Department for Management of Noncommunicable Diseases, Dr. Shanthi Mendis, said that “Early detecting of high blood pressure and lowering heart attack and stroke risk is clearly far less expensive for individuals and governments than heart surgery, stroke care, dialysis, and other interventions that may be needed later if high blood pressure is left unchecked and uncontrolled.” WHO called on all adults around the world to measure their blood pressure to make people know the blood pressure level and take steps to control it on World Health Day 2013 [6]. Some studies have already proved that blood pressure (BP) measurement can significantly control the level of BP, reduce incidence of adverse reactions, decrease the cost of hypertension treatment, and improve adherence to antihypertensive medication [7–11].

Unfortunately, the rate of BP measurement is not optimistic. There are many studies about the current situation of BP measurement. An investigation in Italy found that about 56.9% of hypertensive patients performed one or more BP measurement weekly [12]. In China, about 42.8% hypertensive patients measured BP weekly in Beijing, 36.9% in Chengdu and 46.3% in Guangzhou [13–15]. The low rate of BP measurement needs to be solved quickly.

Since our population is aging, and hypertension affects most elderly people, these individuals are more likely to have organ damage or clinical cardiovascular disease (CVD). In China, the percentage of 65 and older people is 10.1% in 2015, and it predict to reach 26.8% in 2050 [16]. The prevalence of hypertension in China increases with the age [17]. However, the rate of BP measurement among elderly was not optimistic now [13].

In the present study, we focused on the elderly with hypertension in Shandong Province. Shandong Province has the second largest population in China, and the percentage of its elderly population (65+) has reached 13.15% by 2016. What is more, the prevalence of hypertension in Shandong Province was high. In 2013 in Shandong Province, the prevalence of hypertension is 24.33%, higher than most of other provinces [18]. Therefore, Shandong becomes an ideal place to examine the BP measurement among elderly with hypertension and its related factors. Although there have been many studies focused on the associated factors of BP measurement, none of them have explored the differences between rural and urban areas. Our study aims to explore the rural–urban difference of BP measurement among elderly with hypertension and its separate associated factors.

## Methods

### Data and study population

Data were drawn from the fifth Health Service Survey of Shandong Province, which was part of China’s fifth National Health Service Survey (NHSS), conducted in 2013. NHSS is a national representative survey organized and directed by the Ministry of Health of China every 5 years.

To achieve representation of the whole population, a four-stage, stratified, random sampling method was adopted in both years. In the first stage, 20 counties (districts) were randomly selected in Shandong Province. The second stage sampled townships: 100 townships were selected in sampled counties or districts. In the third stage, 200 villages (communities) were selected in sampled townships. In the fourth stage, 12,000 households were identified. For each household, a face-to-face interview was conducted using a structured household questionnaire which was developed by the Center for Health Statistics and Information of the Ministry of Health of China [19–21].

Among a total of 33,070 respondents, we included hypertensive patients aged ≥65 years old (*n* = 2104). After data cleaning (i.e., excluding participants with key variables missing or with logic error answers), a study sample of 2007 hypertensive patients were adopted for analysis.

### Dependent variables

BP measurement frequency was derived from a question “When was the last time you measured your BP?” They could choose one of the five responses (“within a week” “within a month” “within three months” “within six months” “more than six months”). According to Chinese guidelines for the management of hypertension in 2010, patients with controlled BP should measure BP at least once a week [22]. Therefore, we defined measurement frequency as a binary variable: we assigned patients a value of 1 if they have measured BP within 1 week; and for patients who measured BP longer than 1 week, a value of 0 was assigned.

### Independent variables

#### Covariates

Age, gender, and marital status were used as demographic characteristics. Age was categorized as 65 to 69, 70 to 74, 74 to 79, and 80 years or more. Marital status was classified as married and single, latter one includes unmarried, divorced, and widowed. Socioeconomic status was measured separately via education level and personal income level. Education level was classified into three categories: no formal education, primary education, secondary, or above. Personal income was calculated by household income and household size, and classified into 5 levels: 0 to 2500, 2500~5000, 5000~10,000, 10,000~20,000, ≥ 20,000 Yuan, RMB per year. Self-rated health score was categorized as bad, moderate, and good.

#### BP-related variables

BP-related variables included medication frequency, blood pressure level currently, and accepting health care professionals’ guidance. Medication frequency was classified as on time, occasional, and never. BP level currently was categorized as normal and others (abnormal and unsure). The entry “accepting health care professionals’ guidance” was defined by an affirmative response to the question “In recent three months, have you ever been guided by health care professionals in the prevention and treatment of hypertension?”

### Statistical analysis

Data analyses were conducted using SPSS 24.0. Descriptive statistics, including frequency and percentage, were used to describe the social-demographic and BP management characteristics of the patients. Chi-square tests were used to explore the relationships between age, gender, marital status, education level, personal income, self-rated health, BP-related variables, and measurement frequency.

Binary logistic regression models were used to examine the rural–urban difference of measurement frequency. Model 1: univariate model. Model 2: binary logistic regression model, adjusted for demographic characteristics (age, gender, marital status), socioeconomic status (education level and personal income), and self-rated health. Model 3: in addition to the factors in model 2, BP-related variables (medication frequency, BP level currently, accepting health care professionals’ guidance) were included. Univariate models and adjusted binary logistic regression models were used to explore the risk factors of measurement frequency among both rural and urban elderly. Because NHSS has a complex sampling design, sampling weights were considered in the logistic regression analyses. A *P* value of less than 0.05 was considered statistically significant for the test.

## Results

### General characteristic of the participants

Among these 2007 participants, 1107 (55.2%) were rural elderly, 900(44.8%) were urban elderly. The mean age was 72.74 (SD = 6.15), and most of the participants were currently married (72.8%). More than one-third (35%) of the patients had no formal education, and most of them were unemployed (81.1%). As for BP management characteristics, most of the patients took medicine on time (79.1%). More than half of the patients had normal BP currently (58.5%). And approximately two-thirds (69.1%) of them had accepted health care professionals’ guidance. Since weekly BP measurement is more clinically useful, the presentation of results focused on the weekly categories. There was statistically significant difference in the frequency of BP measurement between rural group and urban group. Besides, age, gender, marital status, education level, personal income, self-rated health, medication frequency, BP level currently, and accepting health care professionals’ guidance were also significantly different between rural elderly and urban elderly (Table 1).

**Table 1:** Socio-demographic characteristics of hypertensive elderly in Shandong, China, 2013

Characteristics	Total (*n* = 2007)	Rural (*n* = 1107)	Urban (*n* = 900)	*χ* ^*2*^	*P*
Age				20.159	*< 0.001*
65–69	743(37.0)	417(37.7)	326(36.2)		
70–74	590(29.4)	292(26.4)	298(33.1)		
75–79	373(18.6)	202(18.2)	171(19.0)		
≥ 80	301(15.0)	196(17.7)	105(11.7)		
Gender				15.138	*< 0.001*
Male	850(42.4)	426(38.5)	424(47.1)		
Female	1157(57.6)	681(61.5)	476(52.9)		
Marital status				30.595	*< 0.001*
Single	546(27.2)	356(32.3)	710(78.9)		
Married	1461(72.8)	751(67.8)	190(21.1)		
Education level				421.712	*< 0.001*
No formal education	702(35.0)	561(50.7)	141(15.7)		
Primary education	656(32.7)	386(34.9)	270(30.0)		
Secondary or above	649(32.3)	160(14.5)	489(54.3)		
Employment				319.858	*< 0.001*
Employed	379(18.9)	365(33.0)	14(1.6)		
Unemployed	1628(81.1)	742(67.0)	886(98.4)		
Personal income				749.689	*< 0.001*
0~2500	434(21.6)	408(36.9)	26(2.9)		
2500~5000	353(17.6)	292(26.4)	61(6.8)		
5000~10,000	456(22.7)	245(22.1)	211(23.4)		
10,000~20,000	457(22.8)	127(11.5)	330(36.7)		
≥ 20,000	307(15.3)	35(3.2)	272(30.2)		
Self-rated health				10.715	*0.005*
Bad	708(35.3)	420(37.9)	288(32.0)		
Moderate	905(45.1)	493(44.5)	412(45.8)		
Good	394(19.6)	194(17.5)	200(22.2)		
Medication frequency				47.338	*< 0.001*
On time	1587(79.1)	813(73.4)	774(86.0)		
Occasional	321(16.0)	224(20.2)	97(10.8)		
Never	99(4.9)	70(6.3)	29(3.2)		
Blood pressure level currently				46.105	*< 0.001*
Abnormal/unsure	833(41.5)	534(48.3)	299(33.2)		
Normal	1174(58.5)	573(51.8)	601(66.8)		
Accepting health care professionals’ guidance				12.118	*< 0.001*
Yes	1386(69.1)	800(72.3)	586(65.1)		
No	620(30.9)	306(27.7)	314(34.9)		
Measurement frequency				173.933	*< 0.001*
Weekly	955(47.6%)	380(34.3%)	575(63.9%)		
More than a week	1052(52.42%)	727(65.7%)	325(36.1%)		

Note: The italic entries mean that these results were statistically significant (*P*<0.05)

The result showed that 955(47.6%) elderly patients measured BP weekly and 1052 (52.42%) did not. Among these 955 (47.6%) patients who measured BP weekly, 380 (34.3%) were rural elderly and 575 (63.9%) were urban elderly (Table 2).

**Table 2:** Descriptive statistics for blood pressure measurement frequency

Measurement frequency	Rural (%)	Urban (%)	Total (%)
Weekly	380(34.3)	575(63.9)	955(47.6)
Monthly	445(40.2)	232(25.8)	677(33.7)
3 months	184(16.6)	49(5.4)	233(11.6)
6 months	47(4.2)	24(2.7)	71(3.5)
More than 6 months	51(4.6)	20(2.2)	71(3.5)
Total	1107(55.2)	900(44.8)	2007

### Rural–urban difference in blood pressure measurement in different logistic regression models

The results of logistic regression models for the association between rural–urban group and BP measurement are presented in Table 3. After adjusting for covariates, model 3 showed that rural elderly did BP measurement less than the urban elderly, the OR was 0.467 (95% CI = 0.380–0.575). All of the three models indicated the rural–urban differences in BP measurement frequency (Table 3).

**Table 3:** Factors associated with blood pressure measurement among the hypertensive elderly in Shandong, China, 2013

	Model 1	*P*	Model 2	*P*	Model 3	*P*
	OR (95% CI)		OR (95%CI)		OR (95%CI)	
Residence						
Urban	1.0		1.0		1.0	
Rural	0.292(0.250–0.341)	*< 0.001*	0.459(0.376–0.562)	*< 0.001*	0.467(0.380–0.575)	*< 0.001*
Age						
65–69			1.0		1.0	
70–74			1.062(0.865–1.303)	0.566	1.046(0.849–1.289)	0.672
75–79			0.963(0.769–1.206)	0.743	1.002(0.795–1.262)	0.987
≥ 80			0.839(0.655–1.075)	0.165	0.834(0.647–1.075)	0.162
Gender						
Male			1.0		1.0	
Female			1.053(0.884–1.254)	0.563	1.046(0.874–1.251)	0.626
Marital status						
Single			1.0		1.0	
Married			1.187(0.977–1.441)	0.084	1.236(1.013–1.508)	*0.037*
Education level						
No formal education			1.0		1.0	
Primary education			1.098(0.892–1.352)	0.376	1.135(0.917–1.405)	0.244
Secondary or above			1.397(1.093–1.786)	*0.008*	1.485(1.154–1.911)	*0.002*
Personal income (RMB)						
0~2500			1.0		1.0	
2500~5000			0.940(0.727–1.215)	0.636	0.935(0.719–1.216)	0.616
5000~10,000			1.290(1.005–1.655)	*0.045*	1.274(0.987–1.644)	0.063
10,000~20,000			1.742(1.331–2.279)	*< 0.001*	1.589(1.206–2.093)	*0.001*
≥ 20,000			1.779(1.304–2.428)	*< 0.001*	1.621(1.179–2.229)	*0.003*
Self-rated health						
Bad			1.0		1.0	
Moderate			0.965(0.808–1.152)	0.691	0.877(0.730–1.053)	0.159
Good			0.973(0.778–1.217)	0.812	0.883(0.702–1.111)	0.289
Medication frequency						
On time					1.0	
Occasional					0.503(0.400–0.633)	*< 0.001*
Never					0.371(0.243–0.566)	*< 0.001*
BP^a^ level currently						
Abnormal/unsure					1.0	
Normal					1.292(1.095–1.526)	*0.002*
Accepting health care professionals’ guidance						
Yes					1.0	
No					0.579(0.484–0.693)	*< 0.001*

Note: The italic entries mean that these results were statistically significant (*P*<0.05)

*BP* blood pressure

### Risk factors of blood pressure measurement among rural elderly

The results of logistic regression models showed that personal income was significantly associated with BP measurement frequency among rural elderly. Moreover, elderly hypertensive patients who took medicine occasionally (OR = 0.539, 95%CI = 0.404–0.718) or never took medicine (OR = 0.349, 95% CI = 0.201–0.604) were less likely to practice BP measurement compared to those on time ones. Patients who had not accepted health care professionals’ guidance recently were less likely to engage in timely BP measurement (OR = 0.519, 95% CI = 0.400–0.674) (Table 4).

**Table 4:** Factors associated with blood pressure measurement among rural elderly in Shandong, China, 2013

	Measurement frequency		Univariate model		Adjusted	
	0	1	OR (95% CI)	*P*	OR (95% CI)	*P*
Age					NA^a^	
65–69	260 (62.4)	157 (37.6)	1.0			
70–74	187 (64.0)	105 (36.0)	1.020(0.761–1.366)	0.897		
75–79	140 (69.3)	62 (30.7)	0.871(0.632–1.199)	0.397		
≥ 80	140 (71.4)	56 (28.6)	0.828(0.591–1.160)	0.272		
Gender					NA	
Male	279 (65.5)	147 (34.5)	1.0			
Female	448 (65.8)	233 (34.2)	0.981(0.766–1.257)	0.882		
Marital status					NA	
Single	243 (68.3)	113 (31.7)	1.0			
Married	484 (64.4)	267 (35.6)	1.169(0.902–1.515)	0.238		
Education level					NA	
No formal education	383 (68.3)	178 (31.7)	1.0			
Primary education	247 (64.0)	139 (36.0)	1.165(0.890–1.525)	0.267		
Secondary or above	97 (60.6)	63 (39.4)	1.248(0.872–1.787)	0.225		
Personal income (RMB)						
0~2500	282 (69.1)	126 (30.9)	1.0		1.0	
2500~5000	206 (70.5)	86 (29.5)	0.953(0.716–1.267)	0.739	0.951(0.717–1.261)	0.726
5000~10,000	155 (63.3)	90 (36.7)	1.321(0.984–1.773)	0.064	1.308(0.981–1.745)	0.067
10,000~20,000	63 (49.6)	64 (50.4)	2.039(1.421–2.925)	*< 0.001*	2.064(1.452–2.934)	*< 0.001*
≥ 20,000	21 (60.0)	14 (40.0)	1.372(0.733–2.566)	0.323	1.370(0.739–2.541)	0.318
Self-rated health						
Bad	283 (67.4)	137 (32.6)	1.0		1.0	
Moderate	309 (62.7)	184 (37.3)	1.008(0.790–1.286)	0.949	1.088(0.858–1.380)	0.487
Good	135 (69.6)	59 (30.4)	0.684(0.491–0.951)	*0.024*	0.746(0.540–1.029)	0.074
Medication frequency						
On time	494 (60.8)	319 (39.2)	1.0		1.0	
Occasional	174 (77.7)	50 (22.3)	0.544(0.408–0.727)	*< 0.001*	0.539(0.404–0.718)	*< 0.001*
Never	59 (84.3)	11 (15.7)	0.361(0.207–0.630)	*< 0.001*	0.349(0.201–0.604)	*< 0.001*
BP level currently					NA	
Abnormal/unsure	370 (69.3)	164 (30.7)	1.0			
Normal	357 (62.3)	216 (37.7)	1.183(0.947–1.478)	0.139		
Accepting health care professionals’ guidance						
Yes	492 (61.5)	308 (38.5)	1.0		1.0	
No	235 (76.8)	71 (23.2)	0.513(0.394–0.669)	*< 0.001*	0.519(0.400–0.674)	*< 0.001*

Note: The italic entries mean that these results were statistically significant (*P*<0.05)

*NA* not applicable

*BP* blood pressure

### Risk factors of blood pressure measurement among urban elderly

The results indicated that those married elderly were associated with more frequent BP measurement than those single ones (OR = 1.367, 95% CI = 1.017–1.837). Education levels were significantly associated with the frequency of BP measurement among urban elderly. The results showed that individuals who had secondary or above education (OR = 1.730, 95% CI = 1.217–2.460) were significantly associated with more frequent BP measurement than those individuals who had no formal education. What is more, elderly who reported moderate self-rated health were less likely to measure BP on time than bad ones (OR = 0.743, 95% CI = 0.565–0.977). Those elderly hypertensive patients who took medicine occasionally (OR = 0.426, 95% CI = 0.294–0.619) or never took medicine (OR = 0.374, 95% CI = 0.193–0.725) were less likely to practice BP measurement compared to those who took medicine on time. Besides, patients with normal BP currently were associated with more frequent BP measurement compared to those abnormal/unsure ones (OR = 1.484, 95% CI = 1.154–1.909). Patients who had not accepted health care professionals’ guidance recently were less likely to engage in timely BP measurement compared to those who had (OR = 0.643, 95% CI = 0.500–0.827) (Table 5).

**Table 5:** Binary logistic regression analysis on the risk factors and measurement frequency among urban elderly (*n* = 1107)

	Measurement frequency		Univariate model		Adjusted model	
	0	1	95% CI	*P*	95% CI	*P*
Age					NA	
65–69	118 (36.2)	208 (63.8)	1.0			
70–74	101 (33.9)	197 (66.1)	1.128(0.833–1.528)	0.436		
75–79	61 (35.7)	110 (64.3)	1.171(0.830–1.650)	0.368		
≥ 80	45 (42.9)	60 (57.1)	0.888(0.598–1.319)	0.557		
Gender					NA	
Male	148 (34.9)	276 (65.1)	1.0			
Female	177 (37.2)	299 (62.8)	1.157(0.886–1.511)	0.284		
Marital status						
Single	84 (44.2)	106 (55.8)	1.0		1.0	
Married	241 (33.9)	469 (66.1)	1.406(1.024–1.931)	*0.035*	1.367(1.017–1.837)	*0.038*
Education level						
No formal education	65 (46.1)	76 (53.9)	1.0		1.0	
Primary education	106 (39.3)	164 (60.7)	1.137(0.780–1.657)	0.505	1.144(0.797–1.644)	0.466
secondary or above	154 (31.5)	335 (68.5)	1.714(1.157–2.539)	*0.007*	1.730(1.217–2.460)	*0.002*
Personal income (RMB)					NA	
0~2500	10 (38.5)	16 (61.5)	1.0			
2500~5000	29 (47.5)	32 (52.5)	0.614(0.267–1.415)	0.252		
5000~10,000	85 (40.3)	126 (59.7)	0.731(0.345–1.547)	0.412		
10,000~20,000	114 (34.5)	216 (65.5)	0.828(0.393–1.742)	0.618		
≥ 20,000	87 (32.0)	185 (68.0)	0.921(0.433–0.297)	0.831		
Self-rated health						
Bad	105 (36.5)	183 (63.5)	1.0		1.0	
Moderate	158 (38.3)	254 (61.7)	0.727(0.549–0.963)	*0.026*	0.743(0.565–0.977)	*0.034*
Good	62 (31.0)	138 (69.0)	1.093(0.774–1.544)	0.614	1.121(0.799–1.572)	0.509
Medication frequency						
On time	252 (32.6)	522 (67.4)	1.0		1.0	
Occasional	55 (56.7)	42 (43.3)	0.433(0.297–0.631)	*< 0.001*	0.426(0.294–0.619)	*< 0.001*
Never	18 (62.1)	11 (37.9)	0.378(0.195–0.735)	*0.004*	0.374(0.193–0.725)	*0.004*
Blood pressure level currently						
Abnormal/unsure	194 (32.3)	407 (67.7)	1.0		1.0	
Normal	131 (43.8)	168 (56.2)	1.474(1.143–1.901)	*0.003*	1.484(1.154–1.909)	*0.002*
Accepting health care professionals’ guidance						
Yes	188 (32.1)	398 (67.9)	1.0		1.0	
No	137 (43.6)	177 (56.4)	0.635(0.493–0.819)	*< 0.001*	0.643(0.500–0.827)	*0.001*

Note: The italic entries mean that these results were statistically significant (*P*<0.05)

*NA* not applicable

## Discussion

In our study, 47.6% of elderly patients engaged in weekly or more BP measurement, which is similar with previous studies [16–18, 22]. The data from the HealthStyles survey showed a 57% prevalence of regular use of BP measurement in 2008 for hypertensive adults 65 years of age and older, which was much higher than our results [11, 21].

Our data showed that the prevalence of weekly BP measurement in urban elderly was higher than that in rural elderly (63.9% vs 34.3%), and rural–urban difference was significant in BP measurement frequency after controlling age, gender, marital status, education level, income, self-rated health, and BP-related variables. The first reason is that the knowledge about prevention and treatment of hypertension among patients in rural areas were significantly lower than that in urban areas [23]. Most of the patients in rural areas knew less about the importance of BP measurement and how to measure BP accurately. Second, rural individuals were less likely to own a sphygmomanometer than their counterparts in urban areas [24]. Therefore, BP measurements are poorly accessible in rural areas. Third, the levels of awareness, treatment, and control of hypertension in rural areas were significantly lower than urban areas [6, 7]. Several evidences have suggested that the percentage of participants engaged in weekly BP measurement significantly increased with being aware of, treated for, and controlled for hypertension [25, 26].

Extensive researches showed that BP measurement is the first step of hypertension management, and regular BP measurement contributes to hypertension prevention and control [27]. Our results showed that medication frequency and accepting health care professionals’ guidance were significantly positively associated with BP measurement frequency among both rural and urban elderly. One possible reason may be that the elderly patients with regular medication may pay more attention to BP management and follow doctors’ guidance strictly. These behaviors are very helpful to maintain regular BP measurement. In addition, health care professionals’ guidance may contribute to more frequent engagement in BP measurement. Studies have indicated the importance of professionals’ guidance on BP measurement [26, 28]. Elderly hypertensive patients who had accepted health care professionals’ guidance usually are told to engage in regularly BP measurement. This is much more helpful to assist patients to be aware of the importance of BP measurement.

Among rural elderly, personal income was significantly positively associated with the frequency of BP measurement. Many studies indicated that personal income was an important influencing factor associated with BP control in rural areas, which was in agreement with the finding of this study [29, 30]. The explanation may be that the health service utilization of the low-income patients was lower than that of high-income ones [31]. Patients with high-income have more opportunities to accept the health care professionals’ guidance on BP control and measure blood pressure while receiving the health care service. In that way, they may put more emphasis on BP measurement and measure BP more frequently.

In our study, those married elderly usually practice BP measurement more frequent than single ones in urban area. That may thanks to the reminding of their spouse. What is more, the elderly patients with higher education level engaged in more frequent BP measurement in urban areas. There might be three explanations for this finding. First, patients with high level of education make better use of home sphygmomanometers, which provide convenience for BP measurement [28]. Second, elderly with higher education level usually had higher level of health literacy [32, 33]. There were evidences proved that hypertensive with higher health literacy had better control of BP [34, 35]. That is because that education offers individuals more opportunities to learn about health and health risks; thus, they more likely to choose healthier lifestyle (such as measure BP timely) to prevent or manage diseases [35, 36]. Third, elderly with lower education often face greater socioeconomic stress; therefore, they lack the financial assistance to support BP measurement [37]. Our study also found that hypertension patients with moderate self-rated health were associated with less frequent BP measurement compared to those bad ones. It is mainly because hypertensive whose symptoms were less severe often neglects to take precautions on BP management.

The findings in this report are subject to some limitations. First, the cross-sectional nature of our survey precludes us from assigning cause and effect to our results. Second, the analyses were depended on self-reports, which involves a risk of information bias due to false or inaccurate responses from participants. Third, there is a limitation with assumptions made in converting reported “the last time measure BP” into BP measurement frequency. For example, it is conceivable that a person may measure BP within a week occasionally; however, according to our definition, the person will be classified as engaging in BP measurement weekly. Fortunately, this situation is not common and its impact on the result is not obvious.

## Conclusion

In conclusion, this study found that the elderly in urban areas performed BP measurement more frequent than the elderly in rural areas. In general, personal income, medication frequency, and accepting health care professionals’ guidance were associated factors of BP measurement frequency among rural elderly. Marital status, education level, self-rated health, medication frequency, BP level currently, and accepting health care professionals’ guidance were associated with the BP measurement frequency among urban elderly. The results of this analysis highlight the rural–urban difference in the use of BP measurement and the necessary to better understand the factors associated with more frequent BP measurement. Therefore, more work will be needed for BP measurement to prevent its consequences in rural areas of China. First, the government should develop targeting health promotion policies to improve health literacy among the elderly. Second, the communities should pay close attention to those identified at-risk subgroups to increase the BP measurement frequency among the elderly.

## Abbreviations

BP: Blood pressure
NA: Not applicable
OR: Odds ratio
